# Safety Assessment Systems for Microbial Starters Derived from Fermented Foods

**DOI:** 10.4014/jmb.2207.07047

**Published:** 2022-09-06

**Authors:** Sojeong Heo, Tao Kim, Hong-Eun Na, Gawon Lee, Jung-Hyun Park, Hee-Jung Park, Do-Won Jeong

**Affiliations:** 1 Department of Food and Nutrition, Dongduk Women’s University, Seoul 02748, Republic of Korea; 2 Department of Food and Nutrition, Sangmyung University, Seoul 03016, Republic of Korea

**Keywords:** Starter, traditional fermented food, safety assessment

## Abstract

Microorganisms involved in food fermentation not only improve the aroma and taste of the food, but also enhance its preservation. Thus, they are added as starter cultures to boost the final product quality of commercial fermented foods. Although these microorganisms originate from fermented foods and have a long history of consumption, the European Union recently applied the concept of Qualified presumption of Safety (QPS), which is a safety evaluation system for microorganisms used in food or feed in Europe. The QPS system is a species-level safety system and shares results with the European Novel Food System, a strain-level safety evaluation system. In the United States, microorganisms added to fermented foods are considered as food additives or Generally Recognized as Safe substance. In Korea, food microbe lists are presented at the species level. Moreover, the nation has established a strain-oriented evaluation system that applies temporary safety evaluation methods for food raw materials as well as new raw materials. However, when it comes to microorganisms isolated from traditional fermented foods and other fermented food products, there is no definition of the term “species,” and there is a lack of an evaluation system at the species level. Therefore, such an evaluation system for microbial species used in Korean fermented foods is necessary.

## Fermentation and Microorganisms

During fermentation, macromolecules such as proteins and starch are degraded into amino acids, monosaccharides, organic acids, and volatile compounds by enzymes produced by microorganisms. This contributes to the sensory characteristics of fermented foods, which have improved sensory properties compared with the raw materials [1]. In addition, microorganisms involved in fermentation produce metabolites such as organic acids and bacteriocins, which inhibit the growth of spoilage or pathogenic bacteria, and consequently improve the storage properties of the food [2]. Therefore, fermentation has been used from ancient times as a method of enabling food storage.

Food fermentation can be divided into natural fermentation, back-sloping, and starter fermentation, depending on the origin of the microorganisms involved. Natural fermentation involves microorganisms that are present in the raw materials or the environment. Back-sloping is a method of adding some of the fermented product to the early stages of new fermentations. Starter fermentation is the isolation of pure microorganisms that have successfully led to fermentation and their subsequent use as single or mixed starter strains for new fermentation.

Before the industrial revolution, natural food fermentation was used domestically in Korea, but, as industrialization progressed, mass production systems were introduced. Natural fermentation is unreliable for mass production and can lead to economic losses due to uncertain microbial growth and inconsistent products. To overcome these problems, back-sloping was introduced, although this method opens the possibility of introducing spoilage or harmful bacteria [3]. In starter fermentation, microorganisms derived from fermented foods are clearly identified as a single strain and then applied; starter cultures are intentionally added to the fermentation process to use their enzymes and metabolic capabilities. Starter fermentation is a suitable method for mass production as it can reliably reproduce fermented products, shorten the fermentation period, and inhibit the growth of pathogenic or spoilage bacteria [4]. The form of the starter strains may depend on the desired purpose. Currently, the global food bacteria companies Christian Hansen and DuPont-Danisco are making continuous efforts to discover novel starter candidates for desired fermented foods and consumer-tailored foods.

## Safety Assessment Systems for Starters

The safety of microorganisms involved in fermentation, including starters, has been considered based on the long history of fermented food consumption. In 2010, the European Food and Feed Cultures Association (EFFCA) proposed as a definition for starters the term “food culture,” and defined food cultures as safe, live bacteria, yeast, or fungi used in food production. Various terms such as starter culture, dairy starter, and yogurt starter, can also be applied. In Europe, food cultures are classified as food ingredients, and food cultures used in food must be supported with information in accordance with European Union (EU) regulation 1169/2011.

The importance of evaluating the safety of the microbiota in food cultures has been highlighted. There are two representative safety assessment systems for food cultures: the Qualified Presumption of Safety (QPS) system in the EU, and the Generally Recognized as Safe (GRAS) system in the USA. The EFFCA’s definition of food cultures was announced after the EU introduced and established the QPS concept for microorganisms used in food and feed [5]. Somewhat differently, the GRAS system assesses safety via a long history of use, rather than via an independent safety assessment system.

## QPS System in the EU

Microorganisms that have traditionally been used in food production in Europe have long been used without regulations based on a history of safe consumption. However, microorganisms used as food cultures are intentionally added food raw materials (unlike in natural fermentation), and thus the necessity of safety evaluation was raised. The Novel Food System, which is a safety assessment system for foods that have not previously been used at a significant level in Europe, was developed in 1997. However, it was unclear whether microorganisms that were consumed in conventional fermented foods were included within the scope of the Novel Food System when used as food cultures. In addition, it was undetermined whether microorganisms identified by advances in technology, technology or not, such as newly designated microorganisms of existing species, were included in the scope of the Novel Food System.

**Fig. 1:**
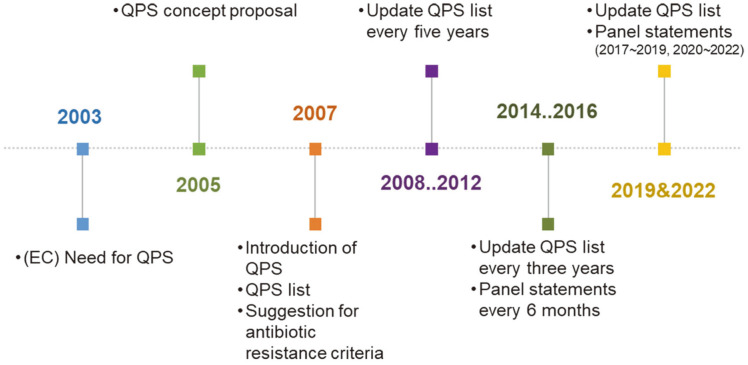
Timeline of development of qualified presumption of safety (QPS) system in European Union .

Experts of the Scientific Committee on Animal Nutrition, the Scientific Committee on Food, and the Plant Science Council on Plants of the European Commission (EC) thus proposed the QPS system concept for the safety evaluation of microorganisms used in food and feed (Fig. 1) [6, 7]. The QPS system applies to microorganisms identified at the species level with a long history of intake and that are present in naturally fermented foods [7].

**Fig. 2:**
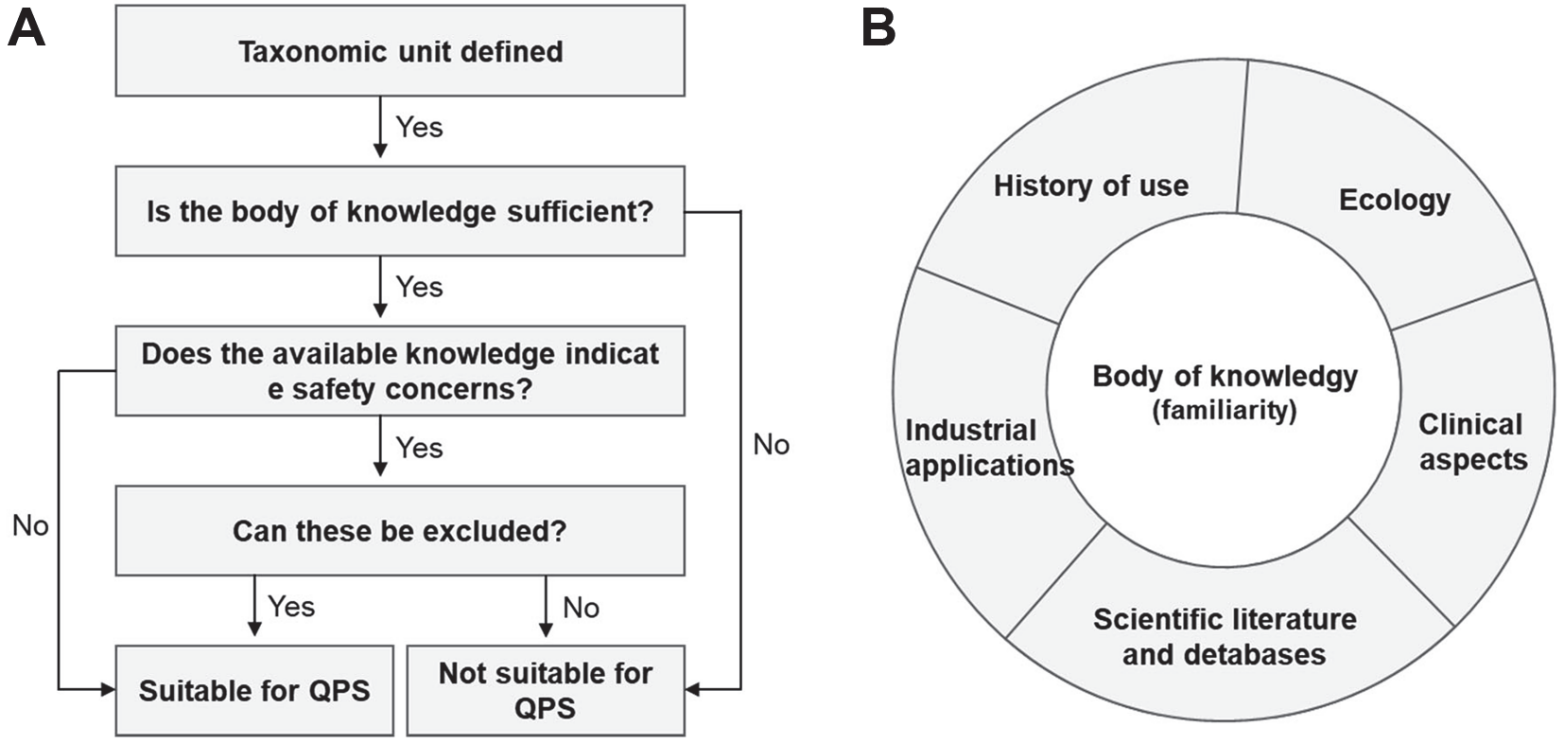
A generalized scheme for assessing the suitability for QPS status of microorganisms (**A**) and components comprising the body of knowledge for assessment (**B**).

The safety evaluation of microorganisms identified at the species level for the QPS system has four aspects (Fig. 2A): taxonomy, body of knowledge (familiarity), pathogenicity, and end-use [7]. The first requirement for approval of a microorganism as having QPS status is that it is clearly classified taxonomically. Therefore, microorganisms should be identified using the most up-to-date methodologies and the most recent nomenclature. Despite this, even if a strain is reclassified into a different species because of the development of technology, its QPS status is not lost. Industrial strains with improved characteristics through mutation and selection are subject to safety evaluation for QPS status. Furthermore, such genetically modified microorganisms require additional review. The second area of evaluation under the QPS system is the body of knowledge about the target microorganism. Since the subjects of QPS evaluation are microorganisms with a long history of use, there should be sufficient quantitative information on them, including any data that may be used to judge their safety, such as the scientific literature and databases, industrial applications, ecology, and clinical aspects (Fig. 2B). Insufficient information related to a microorganism will prevent it from satisfying the QPS requirements. The third area of QPS evaluation is pathogenicity. The microorganism must be unable to cause disease. Some fungi produce mycotoxins, but if they can be deemed safe, strains can gain QPS status. The final criterion for QPS evaluation is end-use. This assesses whether the microorganisms are a component of the final product and will be directly consumed, or whether there are no microorganisms in the final product (i.e., they are only used for production). For example, if safety has been verified for production purposes, the QPS status is only for that purpose.

Microorganisms included in the QPS concept include lactic acid bacteria, *Bacillus* species, yeast, and fungi related to fermented food and feed (Table 1) [7]. These microbial groups were proposed based on the opinions of expert groups [5]. The QPS list was updated annually up to 2012; from 2013, and published every 3 years, but safety issues are published in the form of panel statements every 6 months (Fig. 1) [8-15].

Because microbial units registered in the QPS list have proven general safety, an individual safety assessment is not required if the qualification is not specific, and if the qualification is presented, an assessment of the qualification at the strain level should be conducted. For example, for all members of the genus *Bacillus*, there is a requirement to confirm the absence of toxigenic activity [11, 16]—because food poisoning bacteria such as *B. cereus* belong to this genus, the absence of toxin genes must be shown for a species of *Bacillus* to obtain QPS status. In addition, for all microorganisms, it is necessary to check for the presence or absence of acquired antibiotic-resistance genes [17]. Even if a microbe is on the QPS list, safety evaluation must be conducted at the strain level in accordance with the laws and regulations related to the Novel Food System for use in food. Strains that are not on the QPS list can be applied as Novel Food; however, because they are not species on the QPS list, data should be presented to confirm the safety of such strains.

**Fig. 3:**
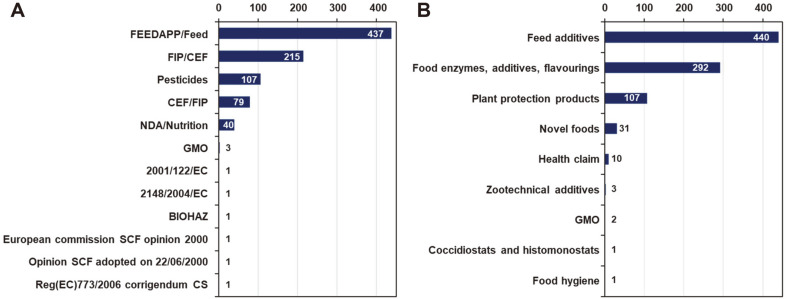
Number of safety assessments by the Biological Hazards (BIOHAZ) panel from other European Food Safety Authority (EFSA) panels or units (**A**), and by EFSA risk assessment area (**B**).

A Biological Hazards (BIOHAZ) panel performs the requested microbial safety assessments for the European Food Safety Authority (EFSA), in addition to updating the QPS list. From 2016 to 2019, there were 887 requests for safety assessment to BIOHAZ, of which 40 cases were requested by the NDA (Panel on Nutrition, Novel Foods and Food Allergens) in charge of Novel Food, and 30 cases were inspected (Fig. 3).

There are many claims that the QPS system is similar to the GRAS system used in the USA. However, the GRAS system is a safety assessment system at the strain level, while the QPS considers safety at the species level, so it is different from the GRAS system, and the Novel Food system bears more similarity to the GRAS system.

## Novel Food System in the EU

The Novel Food System (EC) 258/97 regulation was established in 1997 with the introduction of the Novel Food System, a safety assessment system for food that has never been used at a significant level for human consumption in Europe. Subsequently, in 2015, improvement of the Novel Food Safety Assessment System (EU) 2015/2283 led to changes in clarity of the definition of Novel Foods, centralization of administrative procedures, a transition from individual recognition systems to notification type, and reduction of safety assessment for strains used in other countries.

When an applicant submits a new microbial raw material or microorganism used in the production of new food ingredients, the NDA panel requests evaluation or review by the QPS, and the BIOHAZ panel determines whether the microorganism is on the QPS list or a QPS evaluation target, and informs the NDA panel. Safety assessment of the microorganism can be omitted if it is on the QPS list. If it is not, individual safety assessment should be performed in accordance with the Novel Food System regulations.

Between 2018 and 2020, a total of 46 cases were listed on the EU’s Novel food list, and eight involved microbial raw materials or foods using microorganisms (Table 2). Three involved QPS-listed strains. Four involved E. coli, which is excluded from the QPS because of the complexity of its various disease and pathotoxicity mechanisms [8]. In the remaining case, *Hyphomicrobium denitrificans* strain CK-275 was evaluated as not having QPS status because of a lack of information about it, but the final food product was approved as a Novel Food [18].

As such, if the QPS system is a species-level safety evaluation system based on abundant information and a long history of intake, the Novel Food System can be seen as a component-specific or strain-level safety evaluation system.

## GRAS System in the USA 

In the US, a new food ingredient is recognized as a substance intentionally added to food and is regulated under the Federal Food, Drug and Cosmetics Act (FFDCA). Although new food ingredients must be preapproved by the FDA according to the FFDCA, new food ingredients that are generally considered safe because of a long history of use and evaluation by experts are managed via the GRAS system with manufacturers submitting safety-related data and experts evaluating and notifying them.

In 1958, a list of substances with a long history of intake in the US was published as a GRAS list under the FFDCA. In 1969, President Nixon ordered a review of the safety of GRAS substances and their safety was reviewed for about 10 years from 1972. For substances not on the GRAS list, the GRAS petition (affirmation) system, which involved safety evaluation by the FDA, was operated, but the shortcomings (the long time required and the costs of having the petition approved) were supplemented by converting to the GRAS notification system. The GRAS notification system has been applied since 1997. Notification occurs when the applicant prepares and submits all data and results on the safety of the substance are accepted. Since the system’s introduction, as of December 2020, 937 new food ingredients have been examined, of which 736 were added to the GRAS list. There were 209 newly notified items added to the GRAS list between 2016 and 2020, 26 of which were microorganisms (Table 3). Of these, 24 are function-related microorganisms such as probiotics, and two are genetically modified *S. cerevisiae* for use as starters in alcohol fermentation.

## Fermented Microorganisms and Safety Evaluation Systems in Korea

In Korea, a positive list system (PLS) was introduced and operated from 2016 to provide a list of food ingredients permissible for consumption. A list of microbial raw materials available as food was provided with the introduction of the PLS. However, the list of microorganisms was based on lists of microorganisms used in other countries, so there is a lack of consideration of food microorganisms involved in traditional fermented foods of Korea.

In Korea, common fermented foods include fermented vegetables such as kimchi, foods made with fermented beans such as soybean paste, and fermented fish [19, 20]. Most fermented foods are home-made and based on natural fermentation, but commercial production of fermented foods has increased due to changes in industrialization, housing, family structure and lifestyle, and development of the processed food industry. Because of the increase in industrial production of fermented foods, there is a growing need for fermentation starters. Despite this, the definition and management guidelines regarding starter microorganisms are not clear (although a definition of fungal starters is suggested in the Food Additives Code).

Research on microbial communities in traditional fermented foods has been particularly focused on kimchi, which is produced by the application of starter bacteria. Although the definition of a starter is not clear, a list of lactic acid bacteria involved in the fermentation of dairy products was included in the aforementioned PLS, so these microorganisms can be used as food raw materials. Research on fermented beans has also been conducted actively in recent years, and many results have been obtained from Korea, China, and Japan. Doenjang, soybean paste made using meju, is a high-salt fermented food and salt-resistant bacteria are often predominant in it. Based on recent microbial community analysis of soybean paste through culture-dependent and non-culture-dependent methods, the relationships to the fermentation of bacteria such as *Enterococcus*, *Staphylococcus*, and *Tetragenococcus halophilus* have been reported, alongside members of the genus *Bacillus* [21-23]. Unlike in Europe and the USA, where dairy industries are developed, many Korean fermented foods produced using soybeans have been reported to possess an advantage over fermented soybean products by microorganisms in *Bacillus* [23-27]. A representative microorganism in Korean fermented food is *B. subtilis*. However, with the recent development of analytical techniques, *B. subtilis* has been divided into *B. amyloliquefaciens* subsp. *amyloliquefaciens*, *B. velezensis*, or *B. siamensis* [28]. B. siamensis is not currently on the list of food microbiological ingredients, and it must therefore be registered as a new ingredient.

To be registered as a new food ingredient, relevant documents must be submitted according to the recognition standards for temporary food raw materials (Notification of the Ministry of Food and Drug Safety No. 2020-110). Toxicity test and intake evaluation data must be provided for evaluation; the former must be produced by a Good Laboratory Practice institution, and the experiments are expensive. In the evaluation for the European QPS system, toxicity evaluation data are not required because qualifying organisms have a long history of intake and there is also a large body of knowledge about them. Therefore, compared with the QPS system, the microbial safety evaluation system in Korea is burdened by difficult procedures and high costs.

The dominant bacteria in traditional fermented foods have been documented scientifically on a continual basis, but the application of microorganisms to a species is not easily done. Kimchi is a fermented food produced by lactic acid bacteria, and many of the relevant lactic acid bacteria are registered in Korea as food raw materials. However, microorganisms that are not registered as food raw materials must be registered as temporary food raw materials after a high-cost toxicity evaluation has been conducted.

## Conclusions

Most of the registered microbes among Korea’s food raw materials are those that are also registered as safe in other countries. However, microorganisms involved in fermentation are diverse depending on the raw material and the manufacturing method of the food, so it is not appropriate to compare the microorganisms involved in the traditional fermented foods of Korea with those of other countries. In Europe, members of the genus *Bacillus* have been studied more as plant protection bacteria than as microbes involved in food fermentation. Despite this, in East Asian countries, including Korea, *Bacillus* species are microorganisms involved in fermenting soybean products such as soybean paste, and are valued as starter candidates. Therefore, simplified procedures are needed for commercial starter candidates that have long been consumed in fermented foods. Recently, a coagulase-negative *Staphylococcus* strain was isolated from traditional fermented food but its application is not allowed because it is not registered as a food raw material with the Ministry of Food and Drug Safety. Instead, the strain is a fermented meat species and has a history of overseas use, so it can be used only for fermented meat. In this way, if the use of Korea’s fermented food-derived microorganisms is not possible in Korea due to not having a record of use overseas, it will be difficult to develop or secure novel microbial materials. Therefore, it is necessary to systematically support the application of microorganisms involved in the industrial production of traditional fermented foods in Korea, to enable their broader use.

It is desirable to establish a safety evaluation system in Korea in which food ingredients can be registered as safe if there is sufficient information regarding their long-term intake and use in fermented foods containing a dominant species. In addition, even if a microorganism cannot be registered as a food raw material at the species level, a safety system is required for use in for Korean-style food products. The safety evaluation system needs to approach the safety evaluation considering starter microorganisms, rather than direct ingestion of the microorganisms.

The application of starters is necessary for the globalization of fermented foods and commercial production with uniform quality, and therefore, safety evaluation criteria for starters are required as is evaluation of their functional characteristics. In Europe, the QPS system is a species-level safety evaluation system, and the Novel Food System is a strain-level system. It is also necessary to establish a safety evaluation system in Korea in which species and strains are separated.

## Figures and Tables

**Table 1:** Microorganisms with QPS status in the 2019 list.

**Gram-positive non-sporulating bacteria**			**Gram-positive spore-forming bacteria**	**Gram-negative spore-forming bacteria**	**Yeasts**
*Bifidobacterium adolescentis* ^A^	*Latilactobacillus curvatus* ^A^	*Limosilactobacillus pontis* ^A^	*Bacillus amyloliquefaciens* ^AF^	*Cupriavidus necator* ^AC^	*Limtongozyma cylindracea* ^C^
*Bifidobacterium animalis* ^A^	*Lactobacillus delbrueckii* ^A^	*Limosilactobacillus reuteri* ^A^	*Bacillus atrophaeus* ^AF^	*Gluconobacter oxydans* ^AC^	*Debaryomyces hansenii* ^B^
*Bifidobacterium bifidum* ^A^	*Lapidilactobacillus dextrinicus* ^A^	*Lacticaseibacillus rhamnosus* ^A^	*Niallia circulans* ^ACF^	*Komagataeibacter sucrofermentans* ^AC^	*Hanseniaspora uvarum* ^B^
*Bifidobacterium breve* ^A^	*Lentilactobacillus diolivorans* ^A^	*Latilactobacillus sakei* ^A^	*Alkalihalobacillus clausii* ^AF^	*Xanthomonas campestris* ^AC^	*Kluyveromyces lactis* ^B^
*Bifidobacterium longum* ^A^	*Companilactobacillus farciminis* ^A^	*Ligilactobacillus salivarius* ^A^	*Weizmannia coagulans* ^AF^		*Kluyveromyces marxianus* ^B^
*Carnobacterium divergens* ^A^	*Limosilactobacillus fermentum* ^A^	*Fructilactobacillus sanfranciscensis* ^A^	*Priestia flexa* ^AF^	**Viruses**	*Komagataella pastoris* ^C^
*Corynebacterium ammoniagenes* ^AC^	*Lactobacillus gallinarum* ^A^	*Lactococcus lactis* ^A^	*Lysinibacillus fusiformis* ^AF^	**Plant viruses**	*Komagataella phaffii* ^C^
*Corynebacterium glutamicum* ^AC^	*Lactobacillus gasseri* ^A^	*Leuconostoc citreum* ^A^	*Lederbergia lentus* ^AF^		*Cyberlindnera jadinii* ^C^
*Lactobacillus acidophilus* ^A^	*Lactobacillus helveticus* ^A^	*Leuconostoc lactis* ^A^	*Bacillus licheniformis* ^AF^	*Alphaflexiviridae*	*Ogataea angusta* ^C^
*Companilactobacillus alimentarius* ^A^	*Lentilactobacillus hilgardii* ^A^	*Leuconostoc mesenteroides* ^A^	*Priestia megaterium* ^AF^	*Potyviridae*	*Saccharomyces bayanus* ^B^
*Lactobacillus amylolyticus* ^A^	*Lactobacillus johnsonii* ^A^	*Leuconostoc pseudomesenteroides* ^A^	*Bacillus mojavensis* ^AF^	**Insect viruses**	*Saccharomyces cerevisiae* ^B^
*Lactobacillus amylovorus* ^A^	*Lactobacillus kefiranofaciens* ^A^	*Microbacterium imperiale* ^AC^	*Bacillus paralicheniformis* ^ADF^	*Baculoviridae*	*Saccharomyces pastorianus* ^B^
*Lactobacillus animalis* ^A^	*Lentilactobacillus kefiri* ^A^	*Oenococcus oeni* ^A^	*Bacillus pumilus* ^AF^	**Protists/Algae**	*Schizosaccharomyces pombe* ^B^
*Ligilactobacillus aviarius* ^A^	*Limosilactobacillus mucosae* ^A^	*Pasteuria nishizawae* ^A^	*Bacillus smithii* ^AF^	**Species**	*Wickerhamomyces anomalus* ^C^
*Levilactobacillus brevis* ^A^	*Limosilactobacillus panis* ^A^	*Pediococcus acidilactici* ^A^	*Bacillus subtilis* ^AF^	*Aurantiochytrium limacinum* ^C^	*Xanthophyllomyces dendrorhous* ^B^
*Lentilactobacillus buchneri* ^A^	*Lacticaseibacillus paracasei* ^A^	*Pediococcus parvulus* ^A^	*Bacillus vallismortis* ^AF^	*Euglena gracilis* ^C^	*Yarrowia lipolytica* ^C^
*Lacticaseibacillus casei* ^A^	*Lentilactobacillus parafarraginis* ^A^	*Pediococcus pentosaceus* ^A^	*Bacillus velezensis* ^AEF^	*Haematococcus lacustris* ^C^	*Zygosaccharomyces rouxii* ^B^
*Secundilactobacillus collinoides* ^A^	*Lactiplantibacillus paraplantarum* ^A^	*Propionibacterium acidipropionici* ^A^	*Geobacillus stearothermophilus* ^AF^	*Tetraselmis chuii* ^C^	
*Loigolactobacillus coryniformis* ^A^	*Lactiplantibacillus pentosus* ^A^	*Propionibacterium freudenreichii* ^A^	*Paenibacillus illinoisensis* ^ACF^		
*Lactobacillus crispatus* ^A^	*Lactiplantibacillus plantarum* ^A^	*Streptococcus thermophilus* ^A^	*Parageobacillus thermoglucosidasius* ^ACF^		

Qualification/notes: ^A^The strain should not harbor any acquired antimicrobial resistance genes to clinically relevant antimicrobials; ^B^Absence of resistance to antimycotics used for medical treatment of yeast infections in cases where viable cells are added to the food or feed chain; ^C^QPS applies for “production purposes only” (the qualification “for production purpose only” implies the absence of viable cells of the production organism in the final product and can also be applied for food and feed products based on microbial biomass); ^D^Absence of genetic information to synthesize bacitracin; ^E^Absence of aminoglycoside production ability; FAbsence of toxigenic activity.

**Table 2:** List of approved Novel Foods using microorganisms from 2018 to 2020.

EU No.	Novel Food	Microorganism	QPS
2018/1122	Pyrroloquinoline quinone disodium salt	*Hyphomicrobium denitrificans* CK-275	No
2019/388	2′-Fucosyllactose produced with *Escherichia coli* K12	*Escherichia coli* K12	No
2019/506	D-Ribose	*Bacillus subtilis*	Yes
2019/760	*Yarrowia lipolytica* yeast biomass	*Yarrowia lipolytica*	Yes
2019/1314	Lacto-*N*-neotetraose produced with *Escherichia coli* K12	*Escherichia coli* K12	No
2019/1979	2′-Fucosyllactose/Difucosyllactose mixture	*Escherichia coli* MG1655	No
2020/484	Lacto-*N*-tetraose	*Escherichia coli*	No
2018/1018	UV-treated baker’s yeast	*Saccharomyces cerevisiae*	Yes

**Table 3:** List of notified GRAS substances using microorganisms from 2016 to 2020.

GRN No.	GRAS species/strain	Purpose
660	*Bacillus coagulans* GBI-30	Ingredient in infant formula
670	*Bacillus coagulans* GBI-30	Ingredient in various foods
685	*Lactobacillus plantarum* 299v	Ingredient in conventional foods
722	*Lactobacillus plantarum* Lp-115	Ingredient in various foods
725	*Bacillus coagulans* GBI-30	Ingredient in infant formula
736	*Lactobacillus casei* subsp. *paracasei* Lpc-37	Ingredient in various foods
758	*Lactobacillus helveticus* R0052	Ingredients in infant formula
	*Bifidobacterium longum* subsp*. infantis* R0033	
	*Bifidobacterium bifidum* R0071	
760	*Lactobacillus curvatus* DSM 18775	Antimicrobial agent in ready-to-eat cooked meat and poultry products
798	*Saccharomyces cerevisiae* yBBS002	For use as a starter culture for brewing beer
810	*Lactobacillus paracasei* subsp. *paracasei* F-19e	Ingredient in infant formula
813	*Bifidobacterium longum* BORI	Ingredient in infant formula
814	*Bifidobacterium bifidum* BGN4	Ingredient in infant formula
829	*Aspergillus oryzae*	Ingredient in conventional foods
831	*Bacillus subtilis* DE111	Ingredient in infant formula
840	*Lactobacillus paracasei* F19	Ingredient in conventional foods
841	*Rhizopus oryzae*	For use as a starter culture for brewing beer
847	*Lactobacillus plantarum* ECGC 13110402	Ingredient in various foods
855	*Bifidobacterium animalis* subsp*. lactis* R0421	Ingredient in infant formula
856	*Bifidobacterium animalis* subsp*. lactis* BB-12	Ingredient in conventional foods
864	*Bacillus coagulans* SNZ 1969	Ingredient in infant formula
865	*Lactobacillus acidophilus* NCFM	Ingredient in infant formula
871	*Lactobacillus acidophilus* DDS-1	Ingredient in various foods
872	*Bifidobacterium animalis* subsp. *lactis* UABla-12	Ingredient in various foods.
877	*Bifidobacterium longum* BB536	Ingredient in infant formula
905	*Bacillus subtilis* SG188	Ingredient in conventional foods
949	*Bacillus coagulans* DSM 17654	Ingredient in various foods
